# Development of an Artificial Intelligence Diagnostic System Using Linked Color Imaging for Barrett’s Esophagus

**DOI:** 10.3390/jcm13071990

**Published:** 2024-03-29

**Authors:** Tsutomu Takeda, Daisuke Asaoka, Hiroya Ueyama, Daiki Abe, Maiko Suzuki, Yoshihiro Inami, Yasuko Uemura, Momoko Yamamoto, Tomoyo Iwano, Ryota Uchida, Hisanori Utsunomiya, Shotaro Oki, Nobuyuki Suzuki, Atsushi Ikeda, Yoichi Akazawa, Kohei Matsumoto, Kumiko Ueda, Mariko Hojo, Shuko Nojiri, Tomohiro Tada, Akihito Nagahara

**Affiliations:** 1 Department of Gastroenterology, Juntendo University School of Medicine, Tokyo 113-8421, Japan; psyro@juntendo.ac.jp (H.U.); d-abe@juntendo.ac.jp (D.A.); y.uemura.rn@juntendo.ac.jp (Y.U.); mm-yamamoto@juntendo.ac.jp (M.Y.); t-iwano@juntendo.ac.jp (T.I.); r-uchida@juntendo.ac.jp (R.U.); h-utsunomiya@juntendo.ac.jp (H.U.); s-oki@juntendo.ac.jp (S.O.); nb-suzuki@juntendo.ac.jp (N.S.); at-ikeda@juntendo.ac.jp (A.I.); yakazawa@juntendo.ac.jp (Y.A.); khmatsu@juntendo.ac.jp (K.M.); ktamaki@juntendo.ac.jp (K.U.); mhojo@juntendo.ac.jp (M.H.); nagahara@juntendo.ac.jp (A.N.); 2 Department of Gastroenterology, Juntendo Tokyo Koto Geriatric Medical Center, Tokyo 136-0075, Japan; daisuke@juntendo.ac.jp (D.A.); maikos@juntendo.ac.jp (M.S.); y-inami@juntendo.ac.jp (Y.I.); 3 Department of Medical Technology Innovation Center, Juntendo University School of Medicine, Tokyo 113-8421, Japan; s-nojiri@juntendo.ac.jp; 4 AI Medical Service Inc., Tokyo 171-0013, Japan; tadatomo@ai-ms.com

**Keywords:** artificial intelligence, Barrett’s esophagus, computer-aided diagnosis, linked color imaging, Vision Transformer

## Abstract

**Background:** Barrett’s esophagus and esophageal adenocarcinoma cases are increasing as gastroesophageal reflux disease increases. Using artificial intelligence (AI) and linked color imaging (LCI), our aim was to establish a method of diagnosis for short-segment Barrett’s esophagus (SSBE). **Methods:** We retrospectively selected 624 consecutive patients in total at our hospital, treated between May 2017 and March 2020, who experienced an esophagogastroduodenoscopy with white light imaging (WLI) and LCI. Images were randomly chosen as data for learning from WLI: 542 (SSBE+/− 348/194) of 696 (SSBE+/− 444/252); and LCI: 643 (SSBE+/− 446/197) of 805 (SSBE+/− 543/262). Using a Vision Transformer (Vit-B/16-384) to diagnose SSBE, we established two AI systems for WLI and LCI. Finally, 126 WLI (SSBE+/− 77/49) and 137 LCI (SSBE+/− 81/56) images were used for verification purposes. The accuracy of six endoscopists in making diagnoses was compared to that of AI. **Results:** Study participants were 68.2 ± 12.3 years, M/F 330/294, SSBE+/− 409/215. The accuracy/sensitivity/specificity (%) of AI were 84.1/89.6/75.5 for WLI and 90.5/90.1/91.1/for LCI, and those of experts and trainees were 88.6/88.7/88.4, 85.7/87.0/83.7 for WLI and 93.4/92.6/94.6, 84.7/88.1/79.8 for LCI, respectively. **Conclusions:** Using AI to diagnose SSBE was similar in accuracy to using a specialist. Our finding may aid the diagnosis of SSBE in the clinic.

## 1. Introduction

The frequency of Barrett’s esophagus (BE) and esophageal adenocarcinoma is increasing as the prevalence of gastroesophageal reflux disease (GERD) increases [1]. In Japan, besides the increasing prevalence of GERD, the incidence of BE and esophageal adenocarcinoma is feared to also increase due to an increase in obesity, lifestyle changes, and a decrease in *Helicobacter pylori* infections among the young [2]. In contrast to a diagnosis of long-segment Barrett’s esophagus (LSBE) using white light imaging (WLI), a diagnosis of short-segment Barrett’s esophagus (SSBE) is sometimes more troublesome. Western countries show a high frequency of LSBE. However, in Japan, LSBE is less prevalent than SSBE. After esophagogastroduodenoscopy during health checks, the frequency of SSBE was determined to be from 12.0 to 42.6% on endoscopic screening in Asia [3]. Patients showing LSBE are at higher risk of developing Barrett’s carcinoma; however, patients with SSBE are also susceptible [4]. In Japan, the occurrence of esophageal adenocarcinoma has increased to 6.5% in patients who undergo surgical resections and to 7.5% in those with various types of esophageal cancers, including those who had not undergone a surgical resection [5]. Consequently, an accurate diagnosis of BE is required.

Recently, in a BE diagnosis, esophageal metaplasia (ESEM), as observed by endoscopy, was recognized in the absence of a biopsy [6]. Although magnifying endoscopy was used for a precise diagnosis in several reports [7,8], daily clinical practice requires a diagnosis that is easy to make and accurate. Linked color imaging (LCI) in image-enhanced endoscopy (IEE) allows differences in mucosal color to be easily recognized. As a color-enhancing technology, images from LCI show improved color separation in the red areas of mucosal blood vessels. Such regions are more distinguishable subsequent to the overlap of exposure by narrow-band laser light on a white light laser that maintains the screen’s brightness. As a result, red and discolored lesions are more easily identified, as well as differences in mucosal color. We previously described the usefulness of a diagnosis of BE and reflux esophagitis using LCI [9,10]. Others have described the utility of LCI to aid in the diagnosis of Barrett’s neoplasia and BE [11,12,13,14]; the prevalence of BE using LCI in patients who visited a health center for a detailed medical check-up in Japan was 56.2% [14]. Moreover, as another IEE, the value of texture and color enhancement imaging in evaluating BE has been described [15,16]. It is hoped that IEE will improve the diagnostic capability of BE.

In recent years, artificial intelligence (AI)–based endoscopic diagnostics have been developed and shown to be effective in the field of BE and Barrett’s neoplasia [17,18,19,20,21,22]. The usefulness of using LCI to diagnose *H. pylori* gastritis [23,24,25] and colon polyps [26] was also described. It was noted that adding LCI to AI could improve diagnostic accuracy. However, reports of the diagnosis of BE with LCI using AI are non-existent, and an improvement in the diagnostic performance of BE combining LCI with AI remains unknown. Therefore, we aimed to develop a diagnostic system for BE using AI and LCI.

## 2. Materials and Methods

### 2.1. Preparation of Training and Test Image Sets

Our aim was to develop a diagnostic system for SSBE using an AI system and LCI at a single center in a retrospective clinical study. A total of 624 consecutive patients, treated with esophagogastroduodenoscopy using WLI and LCI (WLI: 696 [SSBE+/− 444/252] and LCI: 805 [SSBE+/− 543/262]) between May 2017 and March 2020 at Juntendo Tokyo Koto Geriatric Medical Center, were retrospectively selected. Equipment used included endoscopic systems (EG-L590WR, EG-L600WR7 or EG-L600ZW; Fujifilm Co., Tokyo, Japan), a light source (LASEREO LL-4450; Fujifilm Co.), and a video processor (AdvanciaHD VP-4450HD; Fujifilm Co.; Structure Emphasis: B6, Color Emphasis: C1). Most study participants were outpatients who were conscious during their endoscopic examination. Endoscopies were carried out for varied reasons: GERD symptoms, a medical check-up, abdominal pain, anemia, and to follow up gastric ulcers. Inclusion criteria of this study were as follows: (1) more than 20 years of age and diagnosed endoscopically as SSBE (SSBE group), or patients who did not have SSBE (control group) and had undergone WLI and LCI; (2) imaging of the gastroesophageal junction (GE-J) occurred during the inspiration of air phase; (3) circumferential images were acquired close to the squamocolumnar junction (SC-J). Exclusion criteria included patients who previously underwent esophageal surgery or a gastrectomy, or who had LSBE, dysplasia, or cancer in SSBE, or advanced esophageal or gastric cancer. Patients were also excluded if it was difficult to perform an endoscopic examination on them due to serious hepatic, respiratory, or heart diseases. In addition, if the GE-J was not fully extended, a patient was also excluded. Obtained images were in JPEG format of acceptable quality. Each image was approximately 100 Kb in size and had a pixel array of 640 × 510 and 24-bit color.

We used expert endoscopists who had each conducted more than 10,000 esophagogastroduodenoscopies (TT1, DA1, MS, YI). An ESEM diagnosis was based on a criterion of BE on endoscopy based on histological confirmation. The GE-J is considered to be at the distal end of the lower esophageal palisade vessels in Japan [27], while the medical establishment in Western countries considers it to be at the proximal end of the gastric folds (Prague C & M criteria) [28]; we used the former definition of GE-J. However, when we could not detect palisade vessels, the GE-J was considered to be at the proximal end of gastric folds. The ESEM was considered to be between the GE-J and SC-J [6]. Two expert endoscopists (TT1, DA1) evaluated WLI endoscopic images until a consensus was reached for each image. Reflux esophagitis was classified according to the Los Angeles (LA) classification system [29], and non-erosive reflux esophagitis was classified according to a modified LA classification system [30,31].

### 2.2. Development of an Endoscopic Diagnosis Support System for Esophageal Barrett’s Mucosa

Images from 528 patients using WLI: 542 (SSBE+/− 348/194) of 696 (SSBE+/− 444/252) or LCI: 643 (SSBE+/− 446/197) of 805 (SSBE+/− 543/262) were used randomly to learn from. A Vision Transformer (Vit-B/16-384, optimizer: “sam”, learning rate: 0.03, batchsize = 64) that diagnosed SSBE was used for two AI systems using WLI and LCI. A Vision Transformer is a neural network that does not use a convolution layer like conventional convolutional neural networks (CNNs) but consists of the encoder part of the transformer (a model that does not use CNNs or recurrent neural networks, but only attention).

### 2.3. Verification of AI Diagnostic Accuracy with Test Data

To assess the accuracy of diagnoses (diagnosis of SSBE or not), a separate test dataset from 96 patients with WLI: 126/LCI: 137 images (SSBE: WLI: 77/LCI: 81, non-SSBE images: WLI: 49/LCI:56) was applied to AI systems. The test dataset used was not augmented. For each test, trained AI systems created a continuous number between 0 and 1 for SSBE or not that corresponded to the probability of a specific condition being represented by an image. The cut-off value of 0.5 was used for a final diagnosis of each condition (SSBE/non-SSBE). Moreover, the area under the receiver-operating characteristic curve (AUC) was calculated in order to assess how accurate the AI-assisted Vision Transformer system was. Accuracy, sensitivity, specificity, positive predictive value (PPV), and negative predictive value (NPV) were determined, respectively. The overall test speed was the period from the beginning to the end of an evaluation of test images as measured by the AI-assisted Vision Transformer system. How the AI-assisted Vision Transformer system recognized input images was assessed using a gradient-weighted class activation map (Grad-CAM) to decide the most important area of each image for classification. The Grad-CAM makes a coarse localization map that highlights pivotal regions in an image to predict a target concept such as SSBE. A heatmap image was created from localization map data.

### 2.4. AI Diagnostic Accuracy Compared to Three Experts and Three Non-Experts

Six endoscopists (three experts, raters A–C: HU1, YA, KM, and three trainees, raters A–C: HU2, TI, MY) assessed whether SSBE was present or not using 263 images (126 WLI and 137 LCI). Accuracy, sensitivity, specificity, PPV, and NPV were determined, respectively. The averages of the three experts and three trainees were evaluated for each value. The images (WLI or LCI) were displayed to each of the endoscopists in a random order independently at a size of 10.3 × 12.9 cm against a black background on a screen (Microsoft Office PowerPoint 2019, Microsoft Inc., Redmond, WA, USA). All images were made anonymous to raters, with no clinical data or dates of images shown.

### 2.5. Statistical Analysis

We undertook statistical analyses using SPSS version 28.0 (SPSS, Inc., Chicago, IL, USA). Continuous data were compared using Student’s *t*-test. Categorical analysis of variables was performed using a Chi-square test or Fisher’s exact test. *p* values < 0.05 were considered significant.

## 3. Results

### 3.1. Patient Characteristics in Training and Validation Datasets

**Figure 1:**
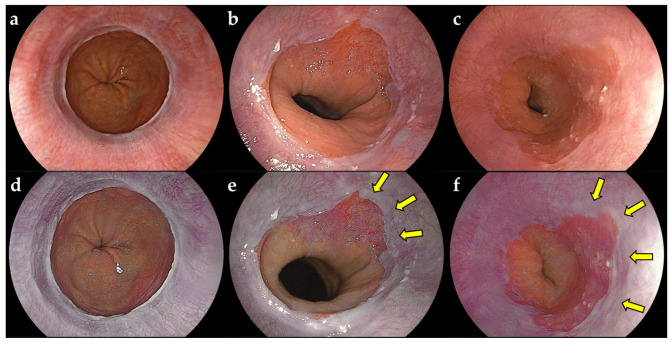
Educational endoscopic images for artificial intelligence diagnostic system using WLI and LCI. (**a**) White light imaging (WLI). Non-short segment Barrett’s esophagus (SSBE) group. (**b**) WLI. SSBE group. Palisade vessels were observed in SSBE. (**c**) WLI. SSBE group. Palisade vessels were not observed in SSBE. (**d**) Linked color imaging (LCI). Non-SSBE group. (**e**) LCI. SSBE group. Yellow arrows indicate SSBE emphasized in a purple color with palisade vessels. (**f**) LCI. SSBE group. Yellow arrows show SSBE emphasized in a purple color without palisade vessels.

The characteristics of the 624 participants of the study are shown in Table 1: mean age (y): 68.7; M/F 330/294; SSBE+/− 409/215. For SSBE, C0M1: 257; C0M2: 38; C1M1: 45; C1M2: 53; C1M3: 5; C2M2: 9; and C2M3: 2 were observed according to Prague C & M criteria. For reflux esophagitis, Grade M: 218; Grade A: 40; Grade B: 13; Grade C: 1; and Grade D: 0 were observed according to a modified LA classification system. Significant differences were not observed in patient characteristics between training and validation data. Figure 1 shows representative cases of non-SSBE and SSBE groups. Short-segment Barrett’s esophagus was emphasized in a purple color using LCI compared to WLI with palisade vessels (Figure 1e). In LCI, SSBE was emphasized in a purple color even when palisade vessels were not noted (Figure 1f).

### 3.2. Accuracy of AI-Assisted Computer-Aided Diagnostic System

**Figure 2:**
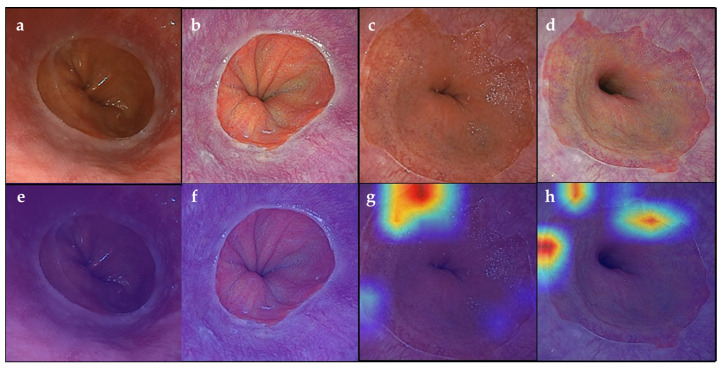
Endoscopic images using WLI and LCI with heatmap images. (**a**) White light imaging (WLI). Non-short segment Barrett’s esophagus (SSBE) group. (**b**) Linked color imaging (LCI). Non-SSBE group. (**c**) WLI. SSBE group. (**d**) LCI. SSBE group. (**e**) WLI. No heatmap was observed. An artificial intelligence (AI) system diagnosed a non-SSBE group. (**f**) LCI. No heatmap was observed. An AI system diagnosed a non-SSBE group. (**g**) WLI. A heatmap was observed. An AI system diagnosed an SSBE group. (**h**) LCI. A heatmap was observed. An AI system diagnosed an SSBE group.

**Figure 3:**
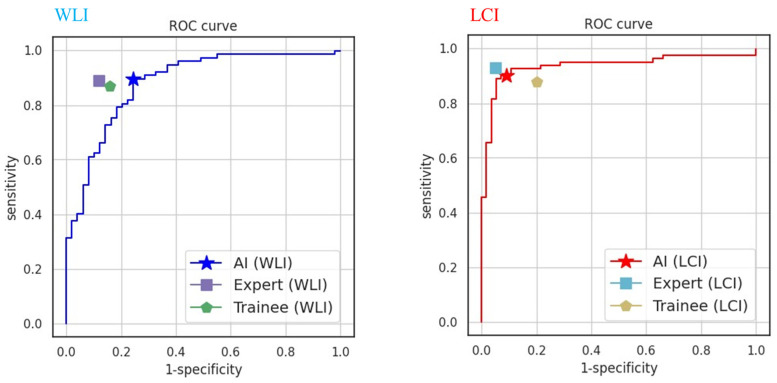
Receiver operating characteristic curve, sensitivity, and specificity of an AI system using WLI and LCI. AI, artificial intelligence; LCI, linked color imaging; ROC, receiver operating characteristic curve; WLI, white light imaging.

Table 2 shows the accuracy of the AI-assisted computer-aided SSBE diagnostic system with WLI and LCI. The scoring of the AI systems with regard to accuracy/sensitivity/specificity/PPV/NPV (%) was 84.1/89.6/75.5/85.2/82.2 for WLI and 90.5/90.1/91.1/93.6/86.4 for LCI. Figure 2 shows heatmap images of the AI system using WLI or LCI. Images that the AI system determined to be SSBE were shown in red. However, images that the AI system judged to not be SSBE were not highlighted in red on the heatmap. Figure 3 shows receiver operating characteristic (ROC) curves of AI, experts, and trainees for WLI and LCI. The area under the curve (AUC) for AI was 0.882 when using WLI and 0.937 when using LCI. Artificial intelligence diagnostic results, with and without palisade vessels or reflux esophagitis, for test data for WLI and LCI are shown in Tables S1 and S2. False negatives were observed in twelve cases when using WLI and in five cases when using LCI. False positives were observed in eight cases when using WLI and in eight cases when using LCI. A significant difference was not observed in terms of accuracy in the presence or absence of palisade vessels and reflux esophagitis when using WLI or LCI. Figure S1 shows heatmap images of false negative and false positive cases.

### 3.3. Diagnostic Accuracy of Endoscopists

Table 3 shows the diagnostic accuracy of trainees and experts when using WLI or LCI. Results for the diagnostic accuracy of experts and trainees with respect to accuracy/sensitivity/specificity/PPV/NPV (%) were 85.7/87.0/83.7/89.3/80.4 for trainees using WLI; 88.6/88.7/88.4/92.3/83.3 for experts using WLI; 84.7/88.1/79.8/86.3/82.2 for trainees using LCI; and 93.4/92.6/94.6/96.2/89.8 for experts using LCI, respectively.

## 4. Discussion

In this study, the development of two AI systems using WLI and LCI rested on a Vision Transformer (Vit-B/16-384) that could diagnose SSBE. Respective findings with regard to the diagnostic accuracy of the AI systems were 84.1% using WLI and 90.5% using LCI. The AI diagnostic system using LCI for SSBE was more accurate compared to using WLI and showed the same accuracy as that of a specialist. For LCI users, adding AI systems to LCI screening may contribute to routine diagnoses. To the best of our knowledge, this is the first study to establish AI systems and the use of LCI for the diagnosis of SSBE.

Linked color imaging is a new technique of IEE and can enhance colors. The exposure of two types of light (short-wavelength narrow-band and white light lasers) is well balanced, which means that data on blood vessels and mucosal surface structures are acquired, as well as information from conventional WLI, simultaneously. The utility of LCI was shown with regard to a marked improved visibility of chronic gastritis [32,33] and the detection of neoplasms in the upper gastrointestinal tract [34] in upper gastrointestinal endoscopy. In recent years, AI systems for the diagnosis of gastritis and early gastric cancer have been developed [35,36,37,38]. In addition, AI systems using LCI have been described. Nakashima et al. reported that an AI diagnostic system for *H. pylori* infection using LCI achieved a sensitivity of 96.7% and a specificity of 83.3% compared to a sensitivity of 66.7% and a specificity of 60.0% using WLI [23]. The diagnostic accuracy for *H. pylori* infection using an LCI–computer-aided diagnostic system was 84.2% for uninfected, 82.5% for currently infected, and 79.2% for post-eradication. An LCI–AI system showed superior diagnostic accuracy compared to that based on a WLI–AI system [25]. Yasuda et al. found that an AI diagnostic system using LCI for the diagnosis of *H. pylori* infection showed an accuracy of 87.6%, a sensitivity of 90.4%, and a specificity of 85.7% [24]. However, regarding BE, the use of LCI in the diagnosis of BE has not been described. A strength of our study is showing the usefulness of an AI system using LCI for SSBE.

This study investigated the accuracy of an AI-assisted and computer-aided SSBE diagnostic system using WLI and LCI. An AI system using LCI showed higher accuracy, sensitivity, specificity, PPV, and NPV than WLI. The usefulness of LCI was also demonstrated by the AI system. This result is similar to that of a previous study on the diagnosis of *H. pylori* infection [23]; the accuracy of an AI system using LCI was higher than that when using WLI. In our previous report [9], SSBE was distinguishable by its purple–red color on LCI and showed improved visibility compared to WLI. An objective evaluation of the color difference (ΔE*) indicated significant differences between the gastric, esophageal, and Barrett’s mucosae. Therefore, when an AI system learns, SSBE is emphasized. This led to an improvement in the diagnosis, even when palisade vessels under WLI were hard to distinguish. Initially, we expected that the accuracy would decrease in the absence of palisade vessels or the presence of reflux esophagitis; however, no significant difference in accuracy was noted between these. An AI system using LCI may have been better because the AI system may have made judgements according to the color of the SSBE, regardless of the presence or absence of palisade vessels. This is considered to be highly useful in clinical practice under various conditions. However, in the evaluation of AI diagnostic results for test data, misdiagnosed cases were observed for both WLI and LCI. According to heatmaps, the locations of the oral or anal sides of the SC-J were not constant in false positive cases (Figure S1). These areas are considered to be areas of interest for AI even if they are not areas that endoscopists would normally diagnose as SSBE. These are characteristics of the AI system, and a further improvement in accuracy, such as by increasing the number of cases studied, is necessary.

The diagnostic accuracy of trainees and experts for SSBE using an AI system and WLI or LCI was also evaluated. Accuracy, sensitivity, specificity, PPV, and NPV were all higher using LCI than when using WLI for both trainees and experts. We previously reported the improved visibility of SSBE using LCI compared to WLI [9]; the higher accuracy for SSBE found with LCI in this current study supports this. Comparing the results of the AI system with those of trainees and experts, the AI system was inferior to experts using WLI but comparable to specialists using LCI. This result is similar to that of a previous report [24] of an AI diagnostic system using LCI for the diagnosis of *H. pylori* infection. A diagnostic AI system for Barrett’s adenocarcinoma has also been reported [17,20,39,40,41]. Better accuracy is expected with the addition of IEEs such as LCI. An AI system using LCI is at about the same level as that of specialists and is expected to have the added effect of equalizing test accuracy and diagnostic accuracy. The use of this system may have a useful impact on improving the quality of clinical endoscopy and SSBE screening in high-risk patients.

Several limitations were apparent in our study. The study was undertaken retrospectively and used a small number of patients from a single center. Second, the AI system that we evaluated did not use video images but only still images, so it could not be applied to a real-time diagnosis. In addition, the AI system does not detect the area of an SSBE but only whether it exists or not. In future, it is necessary to develop AI for an area by annotation. Third, SSBE was not always histologically confirmed, and findings of SSBE by AI were not related to any histological diagnosis since the aim of this investigation was a diagnosis of ESEM only. Furthermore, the study’s Vision Transformer model might have overfit the training set, which would have prevented it from generalizing to previously undiscovered scenarios. To lessen this risk, strategies like regularization and cross-validation could be applied [42]. Therefore, a prospective multi-center study using a bigger cohort with real-time video images and with annotation will be required to further develop our findings. The creation of AI models for the detection of additional esophageal diseases (such as LSBE, dysplasia, or cancer) is recommended for the future.

## 5. Conclusions

We found that an AI diagnostic system using LCI to detect SSBE was as accurate as when using a specialist. Using such an AI system may be useful for making a diagnosis in the esophageal junction area for LCI users in the clinic.

## Figures and Tables

**Table 1:** Baseline characteristics of study patients.

Characteristics	Training Data (n = 528)	Validation Data (n = 96)	*p* Value
Male/female, n	276/252	54/42	0.47
Age, mean (SD), years	68.7 (11.8)	68.6 (11.5)	0.91
SSBE, n present/none	345/183	64/32	0.80
Palisade vessels, n present/none	194/334	35/61	0.96
Reflux esophagitis, n present/none	233/295	39/57	0.52

SD, standard deviation; SSBE, short-segment Barrett’s esophagus.

**Table 2:** Diagnostic accuracy of an AI–assisted computer-aided diagnostic system for SSBE.

	Accuracy	Sensitivity	Specificity	PPV	NPV
WLI	84.1% (106/126)	89.6% (69/77)	75.5% (37/49)	85.2% (69/81)	82.2% (37/45)
LCI	90.5% (124/137)	90.1% (73/81)	91.1% (51/56)	93.6% (73/78)	86.4% (51/59)

AI, artificial intelligence; LCI, linked color imaging; NPV, negative predictive value; PPV, positive predictive value; SSBE: short-segment Barrett’s esophagus; WLI, white light imaging.

**Table 3:** Diagnostic accuracy of endoscopists for SSBE (%).

		Accuracy	Sensitivity	Specificity	PPV	NPV
All endoscopists	WLI	86.3	88.2	83.3	89.1	83.2
	LCI	90.0	91.0	88.6	92.6	88.2
Trainees	WLI	85.7	87.0	83.7	89.3	80.4
	LCI	84.7	88.1	79.8	86.3	82.2
Experts	WLI	88.6	88.7	88.4	92.3	83.3
	LCI	93.4	92.6	94.6	96.2	89.8

LCI, linked color imaging; NPV, negative predictive value; PPV, positive predictive value; SSBE: short-segment Barrett’s esophagus; WLI, white light imaging.

## Data Availability

The datasets used during the present study are available from the corresponding author upon reasonable request.

## Supplementary Materials

The following supporting information can be downloaded at: https://www.mdpi.com/article/10.3390/jcm13071990/s1, Figure S1: Endoscopic images diagnosed as false positive and negative by an AI system using white WLI and LCI with heatmap images; Table S1: Evaluation of AI diagnostic results for test data using WLI; Table S2: Evaluation of AI diagnostic results for test data using LCI.
