# Supplementary material for: Development of an Artificial Intelligence Diagnostic System Using Linked Color Imaging for Barrett’s Esophagus
Source: J Clin Med. 2024 Mar 29;13(7):1990. doi: 10.3390/jcm13071990 (PMC11012507; doi:10.3390/jcm13071990)
Supplement: Supplementary file 1 [file jcm-13-01990-s001.zip › 20240218 TableS1 S2 AI LCI Barrett takeda .pdf]

Table S1. Evaluation of AI diagnostic results for test data using WLI

Parameter			AI diagnosis		total	*P* value	
SSBE	PV	RE	correct n. (%)	incorrect		PV none vs. present	RE none vs. present
+	-	+	18 (90.0)	2	20	0.55	0.13
	+	+	10 (71.4)	4	14		
	-	-	24 (92.3)	2	26		
	+	-	17 (100.0)	0	17		
-	-	+	17 (77.3)	5	22		1
	-	-	20 (74.1)	7	27		
total			106 (84.1)	20	126		

AI, artificial intelligence; PV, palisade vessels; RE, reflux esophagitis; SSBE, short-segment Barrett's esophagus; WLI, white light imaging

Table S2. Evaluation of AI diagnostic results for test data using LCI

Parameter			AI diagnosis		total	*P* value	
						PV	RE
SSBE	PV	RE	correct n (%)	incorrect		none vs. present	none vs. present
+	-	+	16 (80.0)	4	20	0.38	0.25
	+	+	11 (91.7)	1	12		
	-	-	27 (93.1)	2	29		
	+	-	19 (95.0)	1	20		
-	-	+	21 (95.5)	1	22		0.64
	-	-	30 (88.2)	4	34		
total			124 (90.5)	13	137		

AI, artificial intelligence; LCI, linked color imaging; PV, palisade vessels; RE, reflux esophagitis; SSBE, short-segment Barrett's esophagus
