# Supplementary figures and images for: Development of an Artificial Intelligence Diagnostic System Using Linked Color Imaging for Barrett’s Esophagus
Source: J Clin Med. 2024 Mar 29;13(7):1990. doi: 10.3390/jcm13071990 (PMC11012507; doi:10.3390/jcm13071990)

## Slide 1
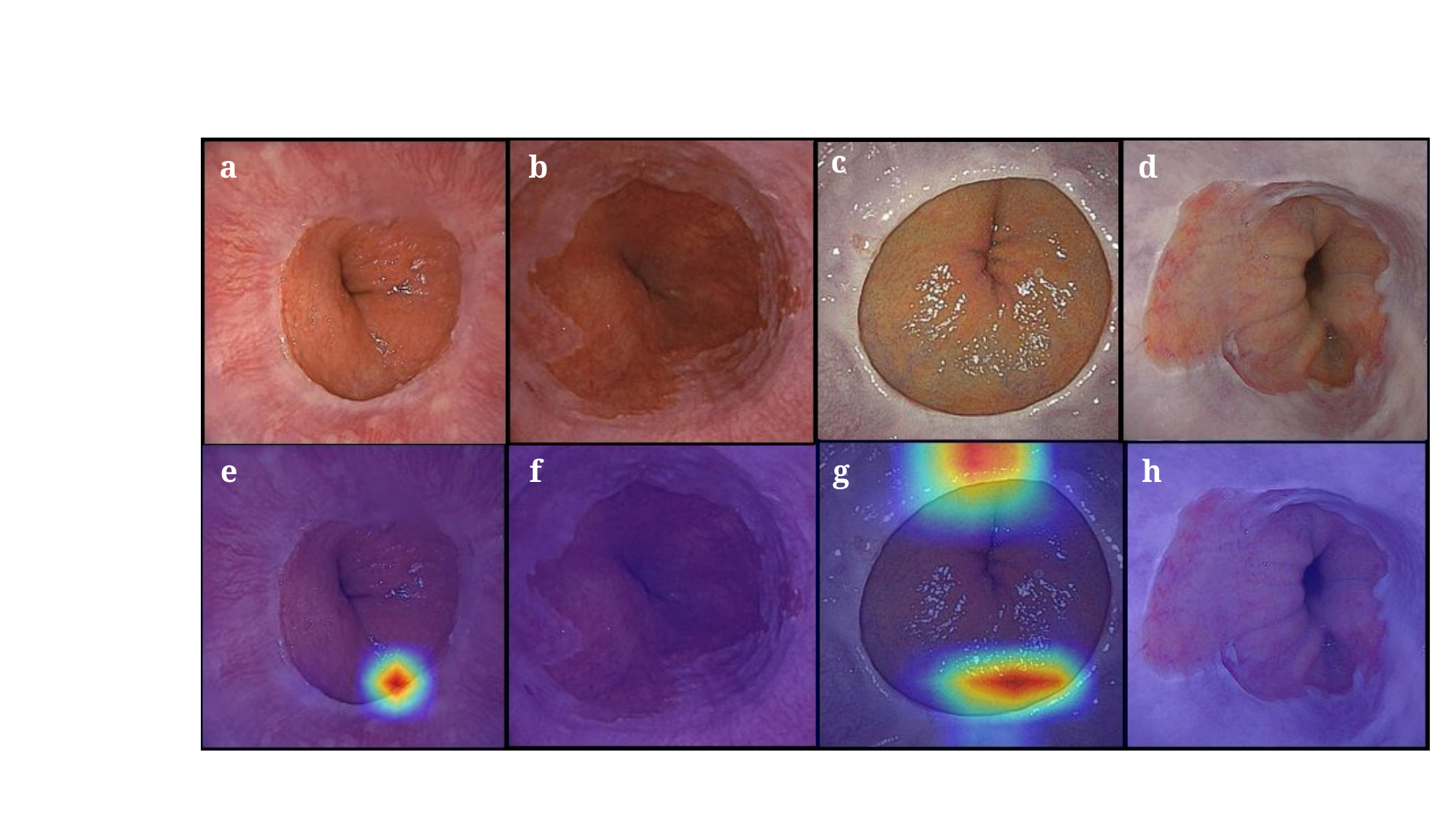

c
a
b
d
e
f
g
h

Supplement: Supplementary file 1 [file jcm-13-01990-s001.zip › 20240218 AI Barrett LCI -Takeda- Figures S1丂.pptx]
